# Robert M. Sanger

**Published:** 1911-07-15

**Authors:** 


ROBERT M. SANGER.—Dr. Sanger died suddenly at his home in East Orange, N. J., April 16th, of arterial schlerosis. Dr. Sanger was a middle-aged man and his death seems untimely. He had been very much interested all his life in Dental Society and kindred professional works. He was, at the time of his death, teaching Prosthetic Dentistry in the College of Dental and Oral Surgery of New York City. He graduated from the New York College of Dentistry in 1891. For thirty years he has been a faithful practitioner in Orange, N. J. and a useful citizen, as he was much interested in all the social and civic affairs of his home town.

